# Supplementary material for: Factors Related to Caregiver Intentions to Vaccinate Their Children with Attention-Deficit/Hyperactivity Disorder against COVID-19 in Taiwan
Source: Vaccines (Basel). 2021 Sep 2;9(9):983. doi: 10.3390/vaccines9090983 (PMC8472816; doi:10.3390/vaccines9090983)
Supplement: Supplementary file 1 [file vaccines-09-00983-s001.zip › vaccines-1361011-supplementary.pdf]

**Supplementary Table S1.: Contents of the Research Questionnaire.**

| Measures | Items | Response scale |
|---|---|---|
| Caregivers' intention to vaccinate their children against COVID-19 | When a COVID-19 vaccine becomes available, will you let your child be vaccinated? | 1 = Definitely willing, 2 = if my doctor recommends it, I would let my child receive it, 3 = not sure, 4 = definitely not willing |
| | Please rate your current willingness to let your child receive a COVID-19 vaccine: | 1 (very low) to 10 (very high) |
| Caregivers' concerns about vaccines | What factors will influence your intention to vaccinate your child against COVID-19? How important will the factors be? (1) Safety of vaccines; (2) effectiveness of vaccines; and (3) family's unfavorable attitude toward child vaccination. | 0 (not important at all) to 3 (very important) |
| Children's use of medication for ADHD | How often does you child take medication prescribed by doctors for ADHD? | 0 = never, 1 = seldom, 2 = sometimes, 3 = often |
| Children's comorbid conduct problems | Before the COVID-19 pandemic, did your child have repetitive and persistent behavioral and emotional problems, such as aggression to people and animals, destruction of property, deceitfulness, lying, or stealing, or serious violations of rules? | 0 = no, 1 = yes |
| Children's comorbid oppositional defiant problems | Before the COVID-19 pandemic, did your child often have angry and resentful mood, argumentative and defiant behavior, or spiteful and vindictive behaviors? | 0 = no, 1 = yes |
| Changes in ADHD and depressive symptoms | Compared with those before the pandemic, did your child's symptoms of (1) inattention, (2) hyperactivity, (3) impulsivity, and (4) depression change during the pandemic? | 0 = improved, 1 = no change, 2 = mildly worsened, 3 = significantly worsened |

ADHD: attention-deficit/hyperactivity disorder; COVID-19 = coronavirus disease 2019.
